# When combat prevents PTSD symptoms—results from a survey with former child soldiers in Northern Uganda

**DOI:** 10.1186/1471-244X-12-41

**Published:** 2012-05-14

**Authors:** Roland Weierstall, Inga Schalinski, Anselm Crombach, Tobias Hecker, Thomas Elbert

**Affiliations:** 1 Department of Clinical Psychology, University of Konstanz, Konstanz, Germany

## Abstract

****Background**:**

Human beings from time immemorial have eradicated neighbouring tribes, languages, religions, and cultures. In war and crisis, the cumulative exposure to traumatic stress constitutes a predictor of the development of post traumatic stress disorder (PTSD). However, homicide has evolved as a profitable strategy in man, leading to greater reproductive success. Thus, an evolutionary advantage of perpetrating violence would be eliminated if the exposure to aggressive acts would traumatize the perpetrator. We argue that perpetrating violence could actually ‘immunize’ a person against adverse effects of traumatic stressors, significantly reducing the risk of developing PTSD.

****Methods**:**

We surveyed 42 former child soldiers in Northern Uganda that have all been abducted by the Lord Resistance Army (LRA) as well as 41 non-abducted controls.

****Results**:**

Linear regression analyses revealed a dose–response effect between the exposure to traumatic events and the Posttraumatic Diagnostic Scale (PDS) sum score. However, the vulnerability to develop trauma related symptoms was reduced in those with higher scores on the Appetitive Aggression Scale (AAS). This effect was more pronounced in the formerly abducted group.

****Conclusions**:**

We conclude that attraction to aggression when being exposed to the victim’s struggling can lead to a substantial risk-reduction for developing PTSD.

## **Background**

It is estimated that at any one time, 300.000 children under the age of 18 are used as soldiers in warfare [1]. Technological advantages like the development of lightweight automatic weapons have resulted in an increase of child soldiers in armed conflicts [2]. From the mid-90’s to 2006 the Lord Resistance Army (LRA) has been fighting in Northern Uganda and is responsible for the mutilation and mass execution of civilians and the abduction and recruitment of children as soldiers in the armed rebel force [3]. Since the LRA relocated its residence to southern Sudan and the Democratic Republic of Congo, several studies have investigated the impact of the war in Northern Uganda and reported devastating effects on the mental health of former child soldiers, especially because of the development of trauma-related symptoms [4-7]. Several studies reported a clear dose-effect relationship between the exposure to different stressful life events and the risk of development of post traumatic stress disorder (PTSD) in various especially war-affected populations [8-11]). Thus, the cumulative exposure to life-threatening events increases the risk of development of PTSD over time. The concept of perpetration-induced traumatic stress (PITS) was introduced to describe an observable phenomenon that former perpetrators show a higher degree of PTSD [12-14]. However, homicide may have evolved as a profitable strategy in man, leading to greater reproductive success and a greater evolutionary benefit of those who perpetrate aggressive acts [15,16]. But such an evolutionary advantage of violent behavior would be eliminated if the exposure to violence cues that are related to their offensive behavior traumatized the perpetrator.

We argue that perpetrating violent acts could actually help to ‘immunize’ a person against adverse effects of traumatic stressors and significantly reduce the risk of developing PTSD [17]. During the course of evolution, not only the purposeful hunting of animals but potentially also of humans may mediate powerful social and psychological rewards and hence interact with the vulnerability of psychological disorders [17]. Most of the research on interspecies aggression stems from primates and has proven that the elimination of neighboring tribes facilitates food supply as well as reproduction and is beneficial for the adults health [18]. Similarly, in-group co-operation and out-group enmity is beneficial for the fitness of the own group in humans [19]. In research on aggression, this instrumental use of aggression for these secondary rewards have been extensively studied. Instrumental aggression has been linked to the secondary rewards, like the gain of social status [20,21], wealth [22], or reproductive success [23]. However, the mutilation of the victims has not been defined as a primary goal of instrumental aggression yet [24,25]. Nell [26] proposed an innate and strongly male-gendered human blood lust that is either expressed by phenomena like bystanders or game hunting in peaceful regions or culminates in the purposeful mutilation of victims in war-affected regions. However, the rewarding effects of cruelty have received only little attention in aggression research. We therefore started to investigate appetitive aggression, i.e. the perpetration of violence and/or the infliction of harm to a victim for the purpose of experiencing violence-related enjoyment, empirically. Recently, we showed in a path-analytic model involving Rwandan prison inmates who were all accused for crimes related to the 1994 genocide that a higher appetitive aggression was associated with a reduced risk to develop trauma-related symptoms [17]. To validate this finding and gain further insight into the underlying pathways that contribute to potential trauma-related illness in perpetrators, we surveyed male former child soldiers that have joined the Ugandan LRA. As we expect a higher degree of attraction to violence in men [26-28] this study focused on male participants. We hypothesized that high levels of appetitive aggression could promote resilience from trauma-related symptoms. The victim’s struggling, their blood and vocalization were defined as the essential rewarding cues for the perpetrator. To control for the impact of socialization the time spend in the bush was added as a covariate to the analyses. Moreover, we supposed that the personally committed delinquency would predict the extend of appetitive aggression.

## **Methods**

### **Participants**

Interviews were carried out with 83 male participants in the age range of 17 to 27 years. The 42 participants of the abducted group have all been formerly abducted by the LRA and spent between 2 days and 12 years in the bush (median = 3 months). To compare our results with a non-abducted control-group, we interviewed 41 young men of comparable age that all have experienced the war in Northern Uganda but who have not spent time as a child soldier in the bush (i.e. less than one day of abduction). Table 1 shows demographic data of the two groups. Chi square tests did not reveal any difference in the observed demographic variables (Chi square tests for age, number of displacements, education and marital status all p > 0.05).

**Table 1:** Demographic Data

	Abducted (n = 42)	Non-Abducted/abducted less than 24 hours (n = 41)
Age, mean (SD), [range]	21.5 (2.47) [17-26]	21.3 (2.51) [18-27]
Age at time of the first abduction	10.7 (3.86) [2-18]	-
No. (%) abducted once abducted more then once*	37 (88.1%)	-
	5 (11.9%)	-
Total duration being abducted (in weeks)	93.5 (153.07) [0.2-624]	-
Period since the last demobilization (mean, (SD), [range] in years)	8.7 (4.2) [1-17]	-
No. (%) of Displacements	1 (2.4%)	0
0	9 (21.4%)	9 (22%)
1	10 (23.8%)	15 (36.6%)
2	8 (19%)	7 (17.7%)
3	13 (31.2%)	9 (22%)
> 4missing	1 (2,4%)	0
Education, No. (%)	13 (31.2%)	15 (36.6%)
No school, some primary school		
Primary school	5 (11.9%)	4 (9.8%)
Vocational School	4 (9.5%)	5 (12.2%)
Some Secondary School	14 (33.3%)	11 (26.8%)
Secondary school	6 (14.3%)	6 (14.6%)
Marital status, No. (%)		
single	24 (57.1%)	25 (61%)
married	6 (14.3%)	5 (12.2%)
partner/cohabiting	12 (28,6%)	10 (24,4%)
divorced	0	1 (2,4%)

*Note. Some participants were abducted more than once, i.e. the LRA sometimes released some of the abducted children after a few days and only used them to carry goods in the bush after a village had been robbed.

The Ethical Review board of the University of Konstanz and the Uganda National Council for Science and Technology approved the study and all participants gave their informed consent. For two participants under the age of 18 the caretakers gave informed consent. Participants received financial compensation of 4,000 USh (Ugandan Shilling). No data set was excluded from the analysis.

### **Procedure**

The survey was conducted in a camp for internally displaced people (IDP) in Pabbo, Northern Uganda. Semi-structured interviews were carried out in Acholi with the help of five local counselors, who have all been trained in the concepts of mental disorders and aggression in a two-month training [29]. Different interpreter translated all questionnaires into Acholi and back into English. The translations were discussed in detail with the interpreters before the application in the interview.

### **Measures**

#### ***Post traumatic symptom severity***

Diagnosis of PTSD and trauma symptom severity in participants was assessed with the Posttraumatic Diagnostic Scale (PDS), validated Acholi version [29,30]. The PDS is a 17-item questionnaire widely used for the diagnosis of PTSD as well as the assessment of PTSD symptom severity. A recent study [31] has proven a good agreement in African conflict populations between the assessment of PTSD symptoms with the PDS and expert clinicians interviews with the CAPS [32], the international gold standard. Each item corresponds to one of the PTSD symptoms specified in DSM–IV and ratings range from 0 (“never”) to 3 (“5 times per week or more/very severe/nearly always”). The PDS assesses the frequency of current PTSD symptoms in the past four weeks related to the most stressful life event. However, the frequency is only rated when the severity of a symptom has clinical significance. Therefore, we used the PDS in a semi-structured clinical interview to assure a valid assessment of PTSD symptoms. For data analysis we used the *PDS sum score*.

#### ***Traumatic event types***

*Traumatic event types experienced* were assessed with a checklist of 34 war- and non-war-related potentially life-threatening events, e.g. injury by weapon, rape, accidents, which also included the events from the PDS [10]. The number of times a specific event had been experienced was not assessed, as distorted memory in PTSD renders this measure unreliable [33-36]. In a previous study in Uganda, the event list showed a high test-retest reliability (r = 0.73, p < 0.001) and significant accordance with the CIDI Event List [29]. For the analysis we further distinguished between the sum of *self-experienced event types*, i.e. events where the life of the participants was in acute danger and the sum of *witnessed event types*, i.e. where the participants witnessed another person in acute life threat.

#### ***Duration in the bush***

As the *duration spent in the bush* could have been a potential predictor for the development of PTSD, we assessed the duration of weeks spend in the bush.

#### ***Appetitive aggression scale***

We assessed a person’s propensity towards perpetrating aggressive acts using the Appetitive Aggression Scale (AAS; [37]), a semi-structured interview for the assessment of a person’s propensity towards violence that has been validated with over 1.600 ex-combatants and that has proven its good psychometric properties. It contains questions regarding the appetitive perception of aggression. Each item is scored on a five-point Likert scale ranging from 0 (“I totally disagree“) to 4 (“I totally agree“). The items are based on the definition of the instrumental aggression subtype according to Vitiello & Stoff [38] and the ICD–10 addiction criteria. Further items were compiled on basis of interviews with perpetrators about the appetitive experience of perpetrating aggressive acts. Cronbach’s Alpha coefficient as a measure for reliability of the Scale was .85. In a principal-axis factoring analysis, all items loaded statistically significant onto a single factor accounting for 32% of the total variance. Moreover, further analyses have revealed that the scale measures a distinct construct of human aggression (for further details see [37]).

#### ***Number of types of offenses***

As a further measure for *self-committed aggressive behavior*, we assessed the number of types of offenses from a list of 16 different types of offenses ranging from physical assault to rape or killings.

#### ***Data analysis***

We used linear regression analysis to explore the association between prognostic variables and the trauma symptom (PDS sum) score. The statistical modeling and analysis was carried out using R for Mac OSX Version 2.11.1 [39].

## **Results**

### **The characteristics of the development of traumatization for both groups**

Only three of the 83 participants (two in the abducted group) fulfilled the full DSM–IV criteria for a PTSD-diagnosis. However, those who were formerly abducted had higher mean PDS sum scores (*M* = 6.6, *SD* = 5.6) than the non-abducted control group (*M* = 3.5, *SD* = 4.9) (*t*(81) = 2.73, *p* = 0.008, *d* = 0.60). Moreover, the two groups differed in the number of experienced as well as witnessed event types, with formerly abducted participants having been exposed to more life-threatening event types (self-experienced event types: *M*_abducted_ = 10.71, *SD*_abducted_ = 3.08; *M*_non-bducted_ = 4.98, *SD*_non-abducted_ = 2.10, *t*(81) = 9.91, *p* < 0.001, *d* = 2.17). (Witnessed event types: *M*_abducted_ = 9.81, *SD*_abducted_ = 2.05; *M*_non-bducted_ = 7.29, *SD*_non-abducted_ = 2.25, *t*(81) = 5.33, *p* < 0.001, *d* = 1.17). The abducted group experienced aggression to be more appetitive than the non-abducted group (AAS sum score: *M*_non-abducted_ = 13.76, *SD*_non-abducted_ = 11.54; *M*_abducted_ = 20.79, *SD*_abducted_ = 15.94, *t*(81) = 2.30, *p* = 0.024).

### **Predictors for trauma related symptoms**

We performed a multiple linear regression analysis to evaluate the factors contributing to the development of trauma symptoms. PDS sum score in the full model was regressed on the number of self-experienced event types, the number of witnessed event types, their squared values, the AAS sum score and all possible two-way interactions. To mitigate multicollinearity, the predictor variables were mean-centered for calculations of interaction terms [40]. For differences in the two sub groups, group was dummy-coded with 0 for non-abducted and 1 for abducted. As stepwise regression analysis is no useful tool for model selection [41], Akaike Information Criterion was used to estimate model fit [42,43]. For the AIC, all possible competing forced entry regression models are ranked according to their AIC, with the one having the lowest AIC being the best fitting, as the loss of information based on this model is the smallest.

The selected model (see Table 2) included the predictors AAS, self-experienced event types, the group dummy, as well as the two interactions self-experienced event types * AAS and group * AAS (*R*_adj_^2^ = 0.43, p < 0.001). The effect sizes for the model as well as the power were calculated using the program G*Power 3 and were sufficient (*f*^2^ = 0.75, (1-) = 1.00) [44]. The model revealed that the number of self-experienced event types was a strong predictor for the development of trauma related symptoms. AAS was no additive predictor. However, as implied by the interplay of the two interactions and the group effect, those who reported an appetitive perception of aggression showed less trauma symptoms, whereby this effect was greater in participants of the abducted group. Moreover, this inverse relation was only crucial as long as the number of self-experienced event types did not exceed a certain threshold.

**Table 2:** Results of regression analyses predicting PDS sum score (N = 83)

	PDS sum score	
	**β**	**p**
self experienced event types	0.87	< 0.001
AAS	−0.01	n.s.
group	- 0.41	0.004
self experienced event types*AAS	0.38	0.025
group*AAS	- 0.36	0.027
R_adj_^2^	0.43	< 0.001

**Note.** Uncorrected standardised regression coefficients are displayed.

**Figure 1:**
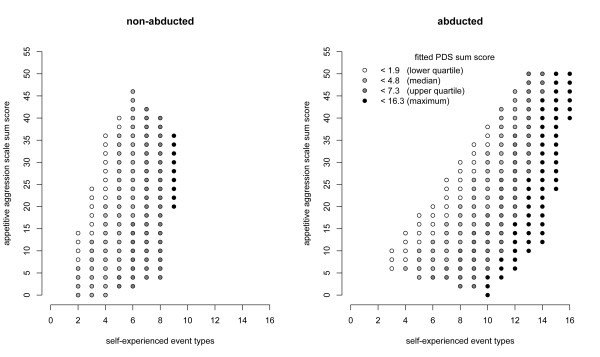
**Fitted PDS sum scores in the non-abducted and the abducted group.** The scores are based on the best fitting linear regression model (see text for details). The grey scale indicates the predicted symptom severity. Only those PDS values are plotted that lie in the convex hulls of the observed predictor values in both groups.

Figure 1 illustrates the interplay between the three predictors. It shows the fitted PDS sum score values that lie in the convex hulls from the observed predictor values in the data sets of the two groups. The two groups are plotted separately for a better graphic representation. The quartiles for the fitted PDS sum scores were calculated from the observed predictor values of the entire sample to enable comparability of the grey scales between the two groups. As can be seen in the abducted group compared to the non-abducted group, there is a near perfect vertical change in grey-scale, i.e., the greater the aggression score, the less the trauma-related symptoms for each class of trauma exposure. However, as can be seen for the abducted group, as trauma exposure increases, ever greater AAS scores are needed, in order to not allow trauma symptoms to develop. Even at high levels of exposure to traumatic stressors there were only two respondents in the abducted group, who actually presented the full clinical picture for PTSD. Consequently, we found a decrease in the vulnerability for trauma related symptoms with an increased duration in the bush, when the correlation between trauma symptoms and the duration in the bush is controlled for the number of self-experienced event types. As Figure 1 also shows, as the number of self-experienced event types increases, the attraction to violence also increases. Interestingly, there are no participants with high number of self-experienced event types but only a low attraction to violence which raises the question to what extent exposure to violence may promote appetitive aggression in the survivor.

Table 3 shows the comparison between the expected PDS sum scores for the two groups, depending on whether a participant would have an AAS sum score and a number of self-experienced event types from the lower or upper quartile of each subgroup. As can be seen, one would await some trauma symptoms to be present in the non-abducted group for the lower quartile of the number of self-experienced event types; either they have a high or low attraction to violence. However, in the abducted group a high AAS sum score would predict that no trauma symptoms should be present, when the number of self-experienced traumatic event types lies at the lower quartile of this group. This effect decreases, when the number lies at the upper quartile, i.e. the number of traumatic event types increases.

**Table 3:** Predicted PDS sum scores for the lower and upper quartiles of the Appetitive Aggression Scale score (AAS) and number of self-experienced event types, depending on group membership

**Non-abducted group**		
	self-experienced event types	
AAS	Lower quartile = 4	Upper quartile = 7
Lower quartile = 4	3.9	6.1
Upper quartile = 25	0 (−0.2)	5.5
**Abducted group**		
	self-experienced event types	
AAS	Lower quartile = 9	Upper quartile = 13
Lower quartile = 7	6.7	9.8
Upper quartile = 38	0 (−0.3)	6.3

**Note.** The fitted PDS sum score for the lower quartile SET and upper quartile AVE in the abducted group falls below 0, which is a result of the underlying model; i.e. one would expect no trauma symptoms (“0”) in these participants.

Further analyses of the residuals revealed that the proposed model fulfills all necessary quality criteria for linear regression analyses. The maximum Variance Inflation Factor (VIF) did not exceed 3.9 so that multicollinearity may be neglected. The residuals did not differ significantly from normal distribution (Kolmogorov–Smirnov *Z* = .97, *p* = .300). Moreover, there was no severe influence of outliers in the proposed models (maximum Cook’s d of .199). However, there was no predictive value of witnessed traumatic event types that could have increased the model fit. To proof that the selected model fitted the data best, we tested whether the second best model according to AIC would improve model fit. However, neither the *R*^2^-change, nor the beta value for the additional predictor (*β*_self experienced event types*group_ = − .72) turned statistically significant (*p* > .05).

Finally, the AAS sum score was regressed on the number of witnessed event types, number of types of offenses, as well as their squared values and all possible two-way interactions. The dummy variable group was added for the analyses of potential group differences. Remarkably, the only significant predictor in all possible models was committed delinquency with an adjusted *R*^2^ of 0.52 (*p* < 0.001), i.e. the reported offences are strongly related to the AAS sum score.

## **Discussion**

In this study, we examined the relationship between appetitive aggression assessed with the AAS and trauma-related symptoms in a sample of formerly abducted child soldiers in Northern Uganda and a non-abducted control group. The Appetitive Aggression Scale [37] assesses the extend of appetitive experience with the perpetration of violence or the infliction of harm that is aimed to experience violence-related enjoyment by the exposure to violence cues such as the struggling of the victim. Appetitive aggression was present at least to some degree in both the abducted and the non-abducted group, except that the extent was more substantial in the formerly abducted group. We identified the number of personally committed crimes to be the best and only significant predictor of AAS sum score in both groups, without significant differences in this relationship between the two groups. This relationship is well explained by assumptions of a cycle of violence: abused children become murderers and perpetrators of other crimes of violence [45,46]. The present results indicate “that violence breeds violence” not simply in the form of reactive aggressive acts but fosters appetitive aggression, which in turn increases the likelihood of violent offenses. Participants in the formerly abducted group experienced more trauma-related symptoms than participants in the non-abducted group which would be expected, given their much greater traumatic stress. However, PTSD related symptoms were reduced or even absent in those who reported violent acts as more positive. The resilience against mental illness in those who report a high propensity towards violence has also been found for perpetrators of the Rwandan genocide [17]. However, there is no ultimate resilience because the protection wanes as the exposure to traumatic stressors exceeds a certain threshold.

Studies on Vietnam Veterans have reported a high degree of PTSD in former soldiers [47]. Even professional torturers reported trauma-associated symptoms and Milgram reported that participants in his study on obedience reported distress when they applied electroshocks [48,49]. Proponents of the PITS-concept suggest that “the human mind… is not well set up for killing” and use the results cited before for their line of argument [12]. We also found a higher PTSD symptom severity in those who perpetrated violence compared to those who were not involved in armed conflicts. However, the conclusion that can be drawn from this result takes on a different connotation when one considers the underlying mechanisms leading to traumatization. Baumeister and Campbell [50] emphasize that “to understand the psychology of perpetrators, it may be necessary to distance oneself from the victim’s view”, as the perception of violence differs remarkably between the perpetrator’s and victim’s perspective [51]. The same seems to be true for the mechanisms leading to the development of trauma-related symptoms. Moreover, to understand why some perpetrators are traumatized while others engage in killings without remarkable distress caused by their own atrocities, we propose that a differentiated view of the perception of cruelty could be crucial. The appeal of violence does not seem to override the strong mechanism of the increasing risk for trauma-related mental illness with a cumulative exposure to threats to one’s life [8,10]. However, it can lead to a substantial risk-reduction. Moreover, it has to be emphasized that only the self-experienced but not the witnessed traumatic event types lead to trauma-related symptoms. Thus, as was indicated by army screening attempts in World War II to sort out those who are vulnerable for “stress disorders”, perpetrators differ in their relative risk of being traumatized [52]. In addition, we would not expect perpetrators to be more affected by the exposure to life-threatening situations in a qualitative manner as suggested by the PITS concept but rather the frequency of the exposure to different life-threatening situations has to be taken into account to draw inference about traumatization in perpetrators. Given the correlational nature of the cross-sectional study, the observed relationship between aggression and traumatic stressors could be also explained by the assumption that those who experience aggression to be more appetitive engage more frequently in severe atrocities. Thus, the likelihood for the exposure to life-threatening situations increases. This explanation is in line with studies that repeatedly demonstrated that the engagement in violence also increases the risk of personal victimization [53-55]. A second explanation can be traced back to the sample characteristics. It has to be emphasized that two types of participants are missing in this sample: Participants with a high number of self-experienced event types and low AAS scores and participants with high AAS scores and a low number of self-experienced event types. We assume that the former ones suffer from more severe PTSD symptoms in line with the model and the risk for PTSD associated mortality would be high too [56,57]. Thus, either this subgroup could not be obtained in a convenient community sample or it became a victim of fatal casualties. Contrary, PTSD symptoms should not be present in the latter ones. We therefore assume that they might still be engaged in fightings of the LRA.

### **Limitations**

We collected the data retrospectively. Even if one third of the population in Uganda experienced the abduction of one of their children [58] and it is estimated that (according to the Survey of War Affected Youths (SWAY)) the Lord’s Resistance Army abducted more than 66,000 children (SWAY 2008 cited in [59]:14), it could be assumed that the LRA very good at selecting more aggressive children so those who stayed in the bush for more than two days were they actually more prone to aggression. Therefore, there could have been differences between the two groups prior to the time in the bush. In would be of great interest for future studies not only to ask for the current attraction to violence but also for the attraction to violence in the period prior to abduction. Another limitation of the sampling that has to be considered concerns the factors involved that compel former child soldiers to escape from the bush. The LRA is still engaged in severe armed conflicts in southern Sudan and the DRC. It could be speculated that those who have a high amount of appetitive aggression and a low number of self-experienced event types, i.e. subgroup in which we would expect trauma-related symptoms to be absent or negligible, are still eagerly involved in manhunt activities and thus unlikely to return to civil society.

## **Conclusions**

The present results confirm a model that suggests that in an environment dominated by organized violence, exposure to traumatic stressors increases appetitive aggression and furthers violent behavior, thus driving a cycle of violence. The increasing appetitive aggression on the other hand seems to relate to a smaller vulnerability for trauma-related mental illness, although there seems to be no ultimate resilience. Therefore, we predict that mental illness will be a likely consequence for those entrapped in the cycle of violence. Future studies have to show if this result can be generalized to other measures of trauma-spectrum disorders.

## **Competing interests**

The author(s) declare that they have no competing interests.

## **Authors’ contributions**

RW: principal investigator, data analysis and manuscript preparation. IS: supervision and training of local psychologists. TH & AC: data analysis and manuscript preparation, local trainers. TE: study design, manuscript preparation and supervisor. All authors read and approved the final manuscript.

## Pre-publication history

The pre-publication history for this paper can be accessed here:

http://www.biomedcentral.com/1471-244X/12/41/prepub
